# Early childhood development and stunting: Findings from the MAL‐ED birth cohort study in Bangladesh

**DOI:** 10.1111/mcn.12864

**Published:** 2019-08-06

**Authors:** Baitun Nahar, Muttaquina Hossain, Mustafa Mahfuz, M. Munirul Islam, Md Iqbal Hossain, Laura E. Murray‐Kolb, Jessica C. Seidman, Tahmeed Ahmed

**Affiliations:** ^1^ Nutrition and Clinical Services Division (NCSD) International Centre for Diarrhoeal Disease Research, Bangladesh (icddr,b) Dhaka Bangladesh; ^2^ Department of Nutritional Sciences The Pennsylvania State University University Park Pennsylvania; ^3^ Division of International Epidemiology and Population Studies Fogarty International Center, National Institutes of Health Bethesda Maryland; ^4^ James P. Grant School of Public Health BRAC University Dhaka Bangladesh

**Keywords:** Bangladesh, children, cognition, early childhood development, MAL‐ED study, stunting

## Abstract

Information on the association between stunting and child development is limited from low‐income settings including Bangladesh where 36% of children under‐ 5 are stunted. This study aimed to explore differences in early childhood development (ECD) between stunted (length‐for‐age *z*‐score [LAZ] < −2) and nonstunted (LAZ ≥ −2) children in Bangladesh. Children (*n* = 265) aged 6–24 months who participated in the MAL‐ED birth cohort study were evaluated by trained psychologists at 6, 15, and 24 months of age using the Bayley Scales of Infant and Toddler Development‐III; child length and weight were measured using standard procedures. ECD scores (*z*‐scores derived from cognitive, motor, language and socio‐emotional skills) were compared between stunted, underweight (weight‐for‐age *z*‐score < −2), and wasted (weight‐for‐length *z*‐score < −2) children, controlling for child age and sex and maternal age, education, body mass index (BMI), and depressive symptoms. Stunted children had significantly lower ECD scores than their nonstunted peers on cognitive (*P* = .049), motor (*P* < .001), language (*P* < .001) and social–emotional (*P* = .038) scales where boys had significantly lower fine motor skills compared with girls (*P* = .027). Mother's schooling and BMI were significant predictors of ECD. Similar to stunting, underweight children had developmental deficits in all domains (cognitive: *P* = .001; fine motor: *P* = .039, and *P* < .001 for both gross motor and total motor; expressive communication: *P* = .032; total language: *P* = .013; social–emotional development: *P* = .017). Wasted children had poor motor skills (*P* = .006 for the fine motor; *P* < .001 for both gross motor and total motor development) compared with the nonwasted peers. Early childhood stunting and underweight were associated with poor developmental outcomes in Bangladesh.

**Key messages**

- Early childhood stunting and underweight at any time point from 6 to 24 months of age are associated with poor cognitive, motor, language and social–emotional skills in Bangladesh.
- Wasted children have poor motor skills compared with their nonwasted peers.
- Stunted boys are deficient in fine motor skills compared with girls.
- Mother's schooling and body mass index are significant predictors of neurocognitive development of the children.

## 1 INTRODUCTION

Globally, stunting (height/length‐for‐age *z*‐score [HAZ/LAZ] below −2 standard deviations (SDs) from the median height‐for‐age of the standard reference population; UNICEF, 2013) is one of the most common manifestations of childhood undernutrition. In Bangladesh, the prevalence of stunting among children under 5 is 36% (National Institute of Population Research and Training (NIPORT), Mitra and Associates, & ICF International, 2016). As an indicator of chronic energy malnutrition, stunting is detrimentally related to both biological and psychosocial developmental milestones. A recent Lancet review (Grantham‐McGregor et al., 2007) on child development clearly shows that for every 10% increase in stunting, the proportion of children reaching the final grade of primary school drops by 8%.

Numerous cross‐sectional and longitudinal studies have documented negative associations between stunting and child development (Black et al., 2013; Grantham‐McGregor et al., 2007). The exact age at which stunting is most significantly related to cognitive difficulty has not been clearly documented. A study in Jamaican children strongly suggests that stunting during the first 2 years of life was related to cognitive deficits in late adolescence (11 out of 12 tests) including deficits on verbal and performance IQ, non‐verbal reasoning, language, and later working memory as well as marked deficits in scores achieved on educational tests including reading and mathematics and a higher drop‐out rate from school (Walker, Chang, Powell, & Grantham‐McGregor, 2005).

Stunted children showed behavioural differences including apathy, more negative effect, and reduced activity, play, and exploration (Aburto, Ramirez‐Zea, Neufeld, & Flores‐Ayala, 2009; Gardner, Grantham‐McGregor, Himes, & Chang, 1999). One Mexican study investigated the activity and exploratory behaviour of infants aged 4–12 months from low socio‐economic status (SES), by direct observation of child behaviour, play, and exploration. The results have shown that lower LAZ and stunting are significantly associated with lower physical activity and lower exploration by the children (Aburto et al., 2009). In another study in Jamaica, the behaviour of stunted and nonstunted children aged 12–24 months was observed in their homes for 2 days during 4 hr of awake time over a period of 6 months. The stunted children showed significantly more apathy, less enthusiasm and variety in exploring, and were less happy and fussier than the nonstunted children. The child behaviour factor score was also significantly lower in the stunted children and associated with locomotor, mental age at enrolment, and with hearing and speech subscale scores at enrolment and after 6 months, on Griffith's Scales of Mental Development (Gardner et al., 1999).

Stunting has been associated with both short‐ and long‐term cognitive and academic performance deficit (Grantham‐McGregor et al., 2007; Kar, Rao, & Chandramouli, 2008; Walker et al., 2005; Walker, Chang, Vera‐Hernandez, & Grantham‐McGregor, 2011) and delayed development of motor skills, which further delays intellectual development and catch‐up physical growth (Mendez & Adair, 1999). There is also evidence that height gain during early childhood is related to cognitive ability even at 12 years of age (Gandhi et al., 2011). In addition, stunted children have vocabulary deficits and other deficiencies in school performance and intelligence (Crookston et al., 2011). The deleterious long‐term effect of stunting was also evident in a recent Indian study that reported that persistently stunted children aged 6 and 12 years had poorer performance on short‐term memory, cognitive speed, retrieval ability, and visuospatial ability tests (Sokolovic et al., 2014).

Although much is known about the association between stunting and cognitive development of children, a knowledge gap persists in lower income countries like Bangladesh. The Etiology, Risk Factors and Interactions of Enteric Infection and Malnutrition and the Consequences for Child Health and Development Study (MAL‐ED) is a multisite project conducted in eight developing countries including Bangladesh (Ahmed et al., 2014). The aim of this project was to gain a better understanding of the risk factors for malnutrition, enteric diseases, and associated health consequences, including cognitive developmental impairment, in children of low‐income countries. Here, we use longitudinal data to compare early childhood development (ECD), that is, cognitive, motor, language, and socio‐emotional skills in stunted and nonstunted (LAZ ≥ −2) children followed from 6 to 24 months of age.

## 2 METHODS

### 2.1 Study design and participants

This was a longitudinal study, using data from the children who participated in the MAL‐ED birth cohort study in Bangladesh (trial registration no: NCT02441426) with information on child development collected at 6, 15, and 24 months of age. Healthy children, living in the urban slum of Dhaka, were enrolled within the first week of birth and followed up to 24 months post‐partum.

Children who suffered from any severe disease requiring hospitalization prior to recruitment and known cases of severe acute or chronic conditions (e.g., neonatal disease, renal disease, chronic heart failure, liver disease, cystic fibrosis, and congenital conditions) were excluded from the study. Children whose mother was <16 years and those who had other siblings already enrolled in the MAL‐ED study were also excluded (Ahmed et al., 2014). Informed written consent was obtained from mothers at the time of enrolment of the children. The survey protocol was approved by the Institutional Review Board of icddr,b.

### 2.2 Study site

The field site of MAL‐ED Bangladesh has been described previously (Ahmed et al., 2014). Briefly, it is located in an urban slum at Bauniabadh area in Section 11 of Mirpur, Dhaka. Similar to other urban slum settings of Bangladesh, the study area is known to have widespread malnutrition and poverty, with women and children bearing most of the burden.

### 2.3 Data collection

#### 2.3.1 Child development

Cognitive, motor, and language development of these children were measured using Bayley Scales of Infant and Toddler Development‐third version (BSID‐III; Bayley, 2005). The BSID‐III is an internationally recognized tool to assess children between the ages of 1 and 42 months, developed by Nancy Bayley in 1969 (Bayley, 2005). The scale consists of a series of developmental play tasks and takes between 45 and 60 min to administer.

The *Cognitive Scale* of the BSID‐III evaluates how a child thinks, reacts, and learns about the world around him or her. It assesses the play skills, attention to familiar and unfamiliar objects, memory, problem‐solving and counting and number skills of a child.

The *Motor Scale* is comprised of two subscales—fine motor and gross motor. The fine motor subscale measures the eye/hand coordination and motor control skills of the hands and fingers such as grasping, holding a spoon, and drawing and the gross motor subscale measures large body complex movements such as sitting, walking, and jumping.

*Language Scale* of the BSID‐III comprised of two subtests: receptive and expressive communication. The receptive communication subtest measures the ability of the child to recognize sounds and understanding spoken words and directions whereas the expressive communication subtest measures the ability of the child to communicate through sounds, gestures, facial expressions or words (simple or difficult), and also assesses how they can combine words into phrases, sentences, and paragraphs.

The *Social–Emotional Scale* assesses emotional and social functioning as well as sensory processing. This scale is administered as a questionnaire to the mothers or caregivers and asks about children's social–emotional milestones that are normally achieved by certain ages: level of interest in colourful or bright things, ease of getting the child's attention or calming the child, ability to take action to get their needs met, use of imagination in play, and how the child uses words to communicate.

The second version of the Bayley scale has been validated (Parveen, Islam, Zaman, Khan, & Ahmed, 2014) and used in previous studies in Bangladesh (Nahar et al., 2009, 2012) and has shown good interobserver reliability and short‐term test–retest stability. BSID‐III has recently been used in several Bangladeshi studies (Aboud, Singla, Nahil, & Borisova, 2013, Singla, Shafique, Zlotkin, & Aboud, 2014), and correlations were run to establish the validity of the subscales (cognitive, language, and fine motor subsets) with age and other predictors (Aboud et al., 2013).

The BSID‐III scale was administered by trained psychologists at 6, 15, and 24 months at the MAL‐ED field site. Three major subsets of BSID‐III (cognitive, motor [fine and gross motor subtests], and language [receptive and expressive language subtests]) and one subtest from mother's or primary caregiver's report (social–emotional) were administered. The raw scores were converted to *z*‐scores.

#### 2.3.2 Nutritional status

Nutritional status was assessed by measuring weight and length of the children by trained field staff. Body weight was measured using Seca 727 Electronic Baby Scale that has a capacity of 20 kg with a precision of 2 g up to 10 kg and 5 g for more than 10 kg. The length was measured using Seca 416 length board that has a range of 33–100 cm (13–39 in.) with 1‐mm accuracy and stature (standing height) in older children. Measurements were taken at a fixed time, preferably morning with minimal clothing and without any shoes. Field workers were also trained to calculate *z*‐scores using new World Health Organization (WHO) growth standards (WHO Multicentre Growth Reference Study Group, 2009).

#### 2.3.3 Maternal measures

Maternal depressive symptoms were assessed using the Self Reporting Questionnaire‐20 (SRQ‐20; Beusenberg & Orley, 1994) by trained field staff. This scale was developed by the WHO for use in developing countries (Harding, De Arango, Baltazar, et al., 1980) and is designed to assess maternal psychological adjustment related to depressive symptoms. The SRQ‐20 is comprised of 20 items or questions, answered with a simple “yes” or “no” (Murray‐Kolb et al., 2014). In this analysis, we used the total score of all 20 questions of SRQ as a continuous measure at 6, 15, and 24 months to correspond with the timing of the Bayley administrations.

The height and weight of mothers were measured 2 months after delivery and body mass index (BMI) was calculated accordingly.

#### 2.3.4 Family characteristics

In the MAL‐ED birth cohort study, information on baseline demographic and socio‐economic characteristics was collected using a questionnaire. The details of the SES of the MAL‐ED birth cohort studies are available elsewhere (Ahmed et al., 2014). In this paper, data on maternal age and education were used. Maternal education was categorized as (a) no schooling, (b) primary incomplete (first to fourth grade), (c) primary complete (fifth to 10th grade), and (iv) higher secondary (11th to 12th grade) and above (graduation and masters).

### 2.4 Outcome measures

The primary outcome measures reported here are the differences in cognitive, motor, language, and social–emotional scores between the stunted and nonstunted children determined at 6, 15, or 24 months of life. The raw scores were converted to *z*‐scores for each time point (at 6, at 15, and at 24 months), referred here as ECD. Bayley assessments were performed late (after the window period of 15 days) in 20 children. Data from these children were included in the final analysis. However, the data from these children did not differ from those measured during the expected window.

### 2.5 Statistical analysis

The child and maternal characteristics at 6 months, that is, at the first time of Bayley administration, were analysed between stunted and nonstunted group. The *z*‐scores of developmental scale (cognitive, motor, language, and social–emotional) at three time points (6, 15, and 24 months) were analysed in longitudinal models. Child and maternal characteristics (maternal age, education, and BMI) compared between groups were analysed using chi‐squared tests for categorical variables and two‐way t‐tests for continuous variables.

The longitudinal models evaluating the associations between stunting/wasting/underweight status and ECD outcomes were adjusted for child age and sex and maternal characteristics including age, schooling, BMI at 2 months post‐partum, and depressive symptoms (SRQ‐20), as these biological and psychosocial factors have been related to child cognitive development (Walker, Wachs, Gardner, et al., 2007).

Generalized estimating equations were used to adjust for repeated measures on each study child (Kung‐Yee & Scott, 1986). The ID of the children was used as the clustering variable. The models were fit using an exchangeable correlation structure and robust standard error was estimated. A *P* value less than .05 was considered statistically significant.

Data were analysed using STATA version 13 (Stata Corp; College Station, Texas, USA).

### 2.6 Ethical consideration

Ethical approval for this study was obtained from the Institutional Review Boards (IRB) of icddr,b. The purpose, methods, and benefits of the study were explained to all mothers before enrolment. The parents had every right to decide for or against participation of their children in the study and also the rights to withdraw their children from the study at any time. Written informed consent of the parents or guardians of the children were taken before enrolment into the study, and confidentiality of all personal information was protected.

## 3 RESULTS

### 3.1 Baseline characteristics

**Figure 1:**
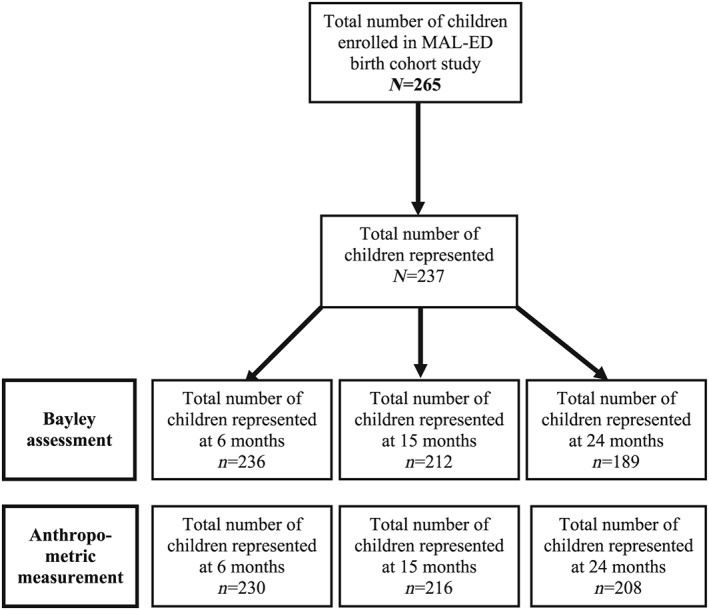
Study flow diagram

A total 265 healthy newborn children living in Mirpur were enrolled in MAL‐ED birth cohort study. The SES of the children was low with suboptimal sanitation, and there were no significantly differences in the SES among the study population. At 6 and 15 months, a total of 236 and 212 children were available, respectively, for testing with the BSID‐III. At 24 months, about 71% of the children (*n* = 189) were assessed for child development. Some BSID‐III assessments were administered late; the maximum day difference between the target date and assessment date was 43 days for 6 months, 79 days for 15 months, and 26 days for 24 months. Figure 1 describes the study flow chart. The major causes of loss to follow‐up were moving out the slum or migrating to another village, unwilling to continue the study, and death.

The median maternal age (interquartile range) was 25 (21–28) years. Around 55% of mothers had attended school for more than 5 years as a part of formal education, whereas 19% never went to school, and only 5% had higher education (secondary and above). Child and maternal characteristics between stunted and nonstunted children at 6 months are described in Table 1. The prevalence of stunting was around 18%, 42%, and 48% at 6, 15, and 24 months, respectively. Children's anthropometric status is shown in Table 2.

**Table 1:** Child's and maternal characteristics between stunted and nonstunted children at 6 months

	Stunted	Nonstunted	*P* value
Child characteristics			
Gender (male) (%)^a^	73.8	43.3	**<.001**
Weight (kg), mean (*SD*)^b^	6.3 (0.9)	7.0 (0.9)	**<.001**
Length (cm), mean (*SD*)^b^	61.4 (2.0)	64.6 (1.8)	**<.001**
Maternal characteristics			
Mother's age, mean (*SD*)^b^	24.7 (5.7)	24.9 (4.8)	.7659
Mother's BMI, mean (*SD*)^b^	22.0 (3.9)	22.5 (3.3)	.3594
Mother's education (%)^a^			
No schooling	23.8	17.1	2.1705
Primary‐incomplete	31	25.7	
Primary‐complete	40.5	52.4	
Higher secondary and above	4.8	4.8	

*Note*. Mother's education: primary incomplete (first to fourth grade); primary complete (fifth to 10th grade); higher secondary (11th to 12th grade) and above (graduation and masters).

Abbreviation: BMI, body mass index.

a Chi‐squared test;

b Two‐way *t*‐test.

The significant values are presented as “bold”

**Table 2:** Anthropometric status of all children from 6 to 24 months

	*N*	WAZ Mean (*SD*)	% Underweight	WHZ Mean (*SD*)	% Wasting	LAZ Mean (*SD*)	% Stunting
6 months	230	−0.9 (1.1)	13	−0.1 (1.0)	4	−1.2 (1.0)	18
15 months	216	−1.4 (1.0)	27	−0.7 (1.0)	11	−1.8 (0.9)	42
24 months	208	−1.6 (1.0)	33	−0.8 (0.9)	9.6	−2.0 (0.9)	48

*Note*. Total number of children represented: 237. Number of children represented: *n* = 230 at 6 months, 216 at 15 months and 208 at 24 months.

Abbreviations: WAZ, weight‐for‐age *z*‐score; WLZ, weight‐for‐length *z*‐score; LAZ, length‐for‐age *z*‐score; stunting, LAZ < −2*SD*; wasting, WLZ < −2*SD*; underweight, WAZ < −2*SD*.

### 3.2 Developmental level

#### 3.2.1 Mean ECD scores of stunted, underweight, and wasted children

**Figure 2:**
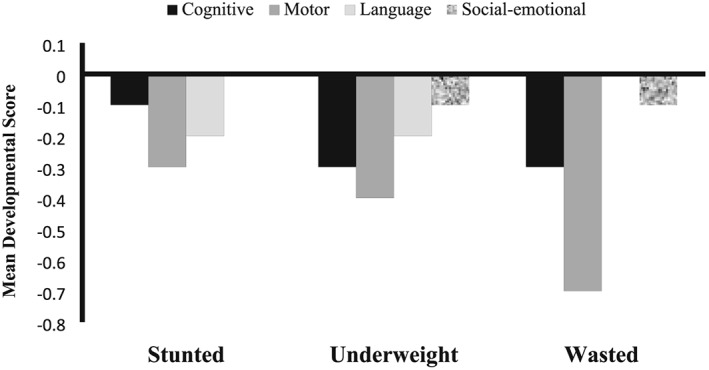
Mean ECD scores (*z*‐scores) of stunted, underweight and wasted children. ECD, early childhood development; stunting: length‐for‐age *z*‐score < −2*SD*; wasting: weight‐for‐length *z*‐score < −2*SD*; underweight: weight‐for‐age *z*‐score < −2*SD*. Stunted: cognitive: 95% CI = −0.16 [−0.31, 0.0], *P* = .049; motor: 95% CI = −0.41 [−0.6, −0.2], *P* < .001; language: 95% CI = −0.37 [−0.56, −0.18]; *P* < .001; and social–emotional: 95% CI = −0.17 [−0.32, −0.0], *P* = .038. Underweight: cognitive: 95% CI = −0.4 [−0.6, −0.2], *P* = .001; motor: 95% CI = −0.6 [−0.8, −0.3], *P* = .039; language: 95% CI = −0.3 [−0.5, −0.1], *P* = .013; and social–emotional: 95% CI = −0.2 [−0.4, −0.04], *P* = .017. Wasted: cognitive: 95% CI = −0.3 [−0.66, 0.03], *P* = .075; motor: 95% CI = −0.77 [−1.1, −0.4], *P* < .001; language: 95% CI = −0.06 [−0.3, 0.2], *P* = .713; and social–emotional: 95% CI = 0.25 [−0.63, 0.13], *P* = .206

The stunted, underweight, and wasted children were equally affected in terms of negative outcomes of ECD scores. The mean *z*‐scores of all developmental domains among stunted, underweight, and wasted children were as follows: (a) cognitive function: −0.1, −0.3, and −0.3; (b) total motor skills: −0.3, −0.4, and −0.7; (c) communication skills: −0.2, −0.2, and 0; and (d) social–emotional skills: 0, −0.1, and −0.1, respectively. The results are shown in Figure 2.

#### 3.2.2 Stunted versus nonstunted children

After controlling for covariates, stunted children had significantly lower developmental scores than the nonstunted children in all domains: cognitive (95% CI = −0.16 [−0.31, 0.0], *P* = .049), motor (95% CI = −0.41 [−0.6, −0.2], *P* < .001), language (95% CI = −0.37 [−0.56, −0.18], *P* < .001), and social–emotional (95% CI = −0.17 [−0.32, −0.0], *P* = .038). These children also experienced *z*‐score deficits on fine motor (95% CI = −0.21 [−0.41, −0.01], *P* = .039), gross motor (95% CI = −0.45 [−0.5, −0.25], *P* < .001), receptive communication (95% CI = −0.3 [−0.49, −0.10], *P* = .003), and expressive communication (95% CI = −0.32 [−0.49, −0.14], *P* < .001) subscales. Boys had significantly lower fine motor skills (95% CI = 0.20 [0.02, 0.38], *P* = .027) compared with girls. No significant difference was observed in the gross motor skill between boys and girls. Interaction or effect modification of the relationship between sex, anthropometry, and ECD was not significant.

Maternal schooling had a significant positive effect on child's development compared with no schooling. Children of mothers who completed primary schooling had significantly higher cognitive (95% CI = 0.33 [0.02, 0.64], *P* = .035), receptive communication (95% CI = 0.36 [0.06, 0.65], *P* = .016), and social–emotional skills (95% CI = 0.48 [0.15, 0.8], *P* = .004) compared with those of mothers with no schooling. Children of mothers who had higher schooling (secondary and above) had significantly higher scores on expressive communication (95% CI = 0.47 [0.07, 0.87], *P* = .02) and total language scores (95% CI = 0.47 [0.06, 0.88], *P* = .024) compared with those of mothers with no schooling. Higher maternal BMI was significantly related to higher motor development of the child (gross motor: 95% CI = 0.04 [0.01, 0.06], *P* = .017; total motor: 95% CI = 0.04 [0.0, 0.06], *P* = .021). No significant effect of maternal depressive symptoms was found on any domains of child development. Results are shown in Table 3.

**Table 3:** Comparison of ECD scores (*z*‐scores)^a^ on BSID‐III between stunted and nonstunted children

	Cognitive				Social–emotional			
Predictors	Unadjusted Coef. [95% CI]	*P* value	Adjusted Coef. [95% CI]	*P* value	Unadjusted Coef. [95% CI]	*P* value	Adjusted Coef. [95% CI]	*P* value
Mother's age	0.0 [−0.01, 0.02]	.847	0.00 [−0.01, 0.03]	.541	−0.02 [−0.04, 0.0]	.183	−0.0 [−0.0, 0.01]	.320
Mother's BMI	0.02 [−0.01, 0.04]	.352	0.00 [−0.02, 0.04]	.520	0.03 [−0.0, 0.06]	.054	−0.0 [−0.03, 0.01]	.528
Mother's education								
No schooling	Reference		Reference		Reference		Reference	
Primary incomplete	0.15 [0.16,0.46]	.335	0.13 [−0.18, 0.44]	.398	0.37 [0.04, 0.71]	.030	0.36 [−0.04, 0.65]	.084
Primary complete	0.30 [−0.03, 0.56]	**.028**	0.33 [0.02, 0.64]	**.035**	0.54 [0.24, 0.84]	**<.001**	0.48 [0.15, 0.8]	**.004**
HSC and above	−0.03 [−0.49, 0.54]	.924	0.06 [−0.5, 0.6]	.828	0.26 [−0.01, 0.67]	.207	0.18 [0.39]	.387
Maternal depressive symptoms	−0.0 [−0.02, 0.02]	.871	0.02 [−0.02, 0.02]	.769	0.0 [−0.02, 0.02]	.872		
Child's age	0 [−0.01, 0.01]	.985	0.00 [−0.0, 0.01]	.365	0.0 [−0.0, 0.0]	.780		
Child's sex (female)	0.06 [−0.1,0.2]	.5	0.09 [−0.1, 0.3]	.805	0.04 [−0.17, 0.25]	.695	0.07 [−0.14, 0.28]	.515
Child's stunting	−0.20[−0.37, −0.03]	**.034**	−0.20 [−0.4, −0.0]	**.049**	−0.16 [−0.31, 0.0]	**.037**	−0.17 [−0.32, −0.0]	**.038**

	Motor											
	Fine motor				Gross motor				Total motor			
Predictors	Unadjusted Coef. [95% CI]	*P* value	Adjusted Coef. [95% CI]	*P* value	Unadjusted Coef. [95% CI]	*P* value	Adjusted Coef. [95% CI]	*P* value	Unadjusted Coef. [95% CI]	*P* value	Adjusted Coef. [95% CI]	*P* value
Mother's age	0.0 [−0.01, 0.02]	.737	−0.0 [−0.01, 0.02]	.963	0.0 [−0.02, 0.03]	.572	0.0 [−0.01, 0.02]	.696	0.0 [−0.01, 0.02]	.63	0.0 [−0.02, 0.03]	.822
Mother's BMI	0.02 [−0.0, 0.05]	.09	0.02 [−0.0,0.05]	.141	0.04 [0.01, 0.06]	**.003**	0.03 [0.0, 0.06]	**.017**	0.04 [0.01, 0.07]	**.005**	0.04 [0.0, 0.06]	**.021**
Mother's education												
No schooling	Reference		Reference		Reference				Reference		Reference	
Primary incomplete	0.08 [−0.1, 0.3]	.509	−0.01 [−0.27. 0.24]	.925	0.17 [−0.12. 0.46]	.262	0.03 [−0.24, 0.31]	.821	0.16 [−0.13, 0.49]	.281	0.02 [−0.26, 0.3]	.899
Primary complete	0.06 [−0.14, 0.27]	.563	0.04 [−0.2, 0.3]	.738	0.24 [0.0, 0.48]	**.042**	0.15 [−0.08, 0.4]	.208	0.22 [−0.02, 0.46]	.071	0.14 [−0.11, 0.41]	.274
HSC and above	−0.0 [−0.5, 0.5]	.977	−0.02 [−0.5, 0.46]	.941	0.17 [−0.14, 0.48]	.272	0.57 [−0.22, 0.33]	.693	0.15 [−0.25, 0.55]	.464	0.06 [−0.03, 0.41]	.757
Maternal depressive symptoms	−0.0. [−0.02, 0.01]	.428	−0.0 [−0.02, 0.01]	.498	−0.01 [−0.02, 0.01]	.429	‐		−0.0 [−0.03, 0.01]		−0.0 [−0.2, 0.01]	.607
Child's age	0.0 [−0.0, 0.01]	.428	0.0 [−0.00. 0.02]	.368	0.0 [−0.0, 0.01]	.949	0.0 [−0.0, 0.02]	.821	0.0 [−0.0 [0.0]	.988	00. [−0.0, 0.01]	.102
Child's sex (female)	0.20 [0.05, 0.4]	.428	0.20 [0.02, 0.38]	**.027**	0.02 [−0.2, 0.2]	.858	−0.04 [−0.2, 0.1]	.208	0.11 [−0.08, 0.3]	.268	0.06 [−0.14, 0.26]	.564
Child's stunting	−0.23 [−0.42, −0.05]	.428	−0.21[−0.41, −0.01]	**.039**	−0.42 [−0.6, −0.24]	**<.001**	−0.45 [−0.5, −0.25]	.693	−0.39 [−0.57, −0.21]	**<.001**	−0.41 [−0.6, −0.2]	**<.001**

	Language											
	Receptive communication				Expressive communication				Total language			
Predictors	Unadjusted Coef. [95% CI]	*P* value	Adjusted Coef. [95% CI]	*P* value	Unadjusted Coef. [95% CI]	*P* value	Adjusted Coef. [95% CI]	*P* value	Unadjusted Coef. [95% CI]	*P* value	Adjusted Coef. [95% CI]	*P* value
Mother's age	−0.0 [−0.03, 0.0]	.371	−0.0 [−0.02, 0.02]	.255	−0.0 [−0.02, 0.01]	.651	−0.00 [−0.03, 0.01]	.456	−0.0 [−0.02, 0.01]	.498	−0.0 [−0.03, 0.13]	.503
Mother's BMI	0.02 [−0.01, 0.04]	.236	0.0 [−0.02, 0.35]	.536	0.02 [−1.01, 0.12]	.115	0.018 [0.0, 0.04]	.160	0.02 [−0.0, 0.04]	.132	0.01 [−0.01, 0.04]	.231
Mother's education												
No schooling	Reference		Reference		Reference		Reference		Reference		Reference	
Primary incomplete	0.16 [−0.11, 0.43]	.251	0.14 [−0.15, 0.43]	.350	−0.0 [−0.28, 0.26]	.928	−0.11 [−0.38, 0.16]	.408	0.03 [−0.02, 0.3]	.824	−0.04 [−0.32, 0.23]	.762
Primary complete	0.35 [0.09, 0.6]	**.006**	0.36 [0.06, 0.65]	**.016**	0.15 [−0.08, 0.39]	.193	0.07 [−0.17, 0.31]	.552	0.26 [0.02, 0.51]	**.037**	0.22 [−0.05, 0.49]	.111
HSC and above	0.3 [−0.14, −0.75]	.178	0.33 [−0.13, 0.8]	.162	0.56 [0.15, 0.94]	**.007**	0.47,[0.07, 0.87]	**.020**	0.5 [0.07, 0.93]	**.021**	0.47 [0.06, 0.88]	**.024**
Maternal depressive symptoms	0.0 [−0.0, 0.02]	.326	‐	‐	0.0 [−0.01, 0.02]	.984	‐	‐	0. [−0.01, 0.02]	.544	‐	‐
Child's age	−0.0 [−0.01, 0.0]	.934	0.0 [−0.0, 0.01]	.255	−0.00 [−0.01, 0.01]	.981	0.0 [−0.0, 0.02]	.210	−0.0 [−0.01, 0.0]	.943	0.0 [−0.0, 0.01]	.154
Child's sex (female)	0.14 [−0.04, 0.32]	.136	0.17 [−0.02, 0.36]	.092	0.07 [−0.1, 0.25]	.414	0.06 [−0.11, 0.25]	.489	0.13 [−0.05, 0.31]	.153	0.14 [−0.04, 0.33]	.135
Child's stunting	−0.30 [−0.48, −0.11]	**<.001**	−0.3 [−0.49, −0.10]	**.003**	−0.31 [−0.48, −0.13]	**.001**	−0.32 [−0.49, −0.14]	**<.001**	−0.4 [−0.54, −0.18]	**<.001**	−0.37 [−0.56, −0.18]	**<.001**

*Note*. Mother's education: primary incomplete (first to fourth grade); primary complete (fifth to 10th grade); HSC = higher secondary (11th to 12th grade) and above (graduation and masters). Number of observation: 637; number of children represented: 237; number of children represented: *n* = 236 at 6 months, 212 at 15 months and 189 at 24 months. Reference: base score against which the others were compared.

Abbreviations: BMI, body mass index; BSID‐III, Bayley Scales of Infant Development‐third version; ECD, early childhood development; stunting, length‐for‐age *z*‐score < −2*SD*; nonstunting, length‐for‐age *z*‐score ≥ −2*SD*.

a Generalized estimating equation analysis controlling for age and sex of the child and mother's age, schooling, BMI, and depressive symptoms. Standard error adjusted for clustering on ID.

The significant values are presented as “bold”

#### 3.2.3 Underweight versus not underweight children

Similar to stunting, underweight children had significantly poorer cognitive (95% CI = −0.4 [−0.6, −0.2], *P* = .001), motor (fine motor: 95% CI = −0.6 [−0.8, −0.3], *P* = .039; gross motor: 95% CI = −0.6 [−0.8, −0.4], *P* < .001; and total motor: 95% CI = −0.5 [−0.8, −0.3], *P* < .001), language (expressive communication: 95% CI = −0.2 [−0.4, −0.01], *P* = .032; total language: 95% CI = −0.3 [−0.5, −0.1], *P* = .013; approached to significance with receptive communication skills: 95% CI = −0.2 [−0.4, 0.0], *P* = .055), and social–emotional development (95% CI = −0.2 [−0.4, −0.04], *P* = .017) compared with the children who were not underweight. Boys had significantly lower fine motor skills (95% CI = 0.02 [0.05, 0.4], *P* = .011) and receptive communication (95% CI = 0.2 [0.01, 0.4], *P* = .037) compared with girls after adjusting for underweight status and maternal factors (Table S1).

#### 3.2.4 Wasted versus nonwasted children

Wasted children had significantly poorer motor (fine motor: 95% CI = −0.37 [−0.6, −0.1], *P* = .006; gross motor: 95% CI = −0.73 [−1.1, −0.4], *P* < .001; total motor development: 95% CI = −0.77 [−1.1, −0.4], *P* < .001) skills relative to nonwasted children. Boys had significantly lower performance compared with girls in fine motor (95% CI = 0.24 [0.06, 0.4], *P* = .007), receptive communication (95% CI = 0.2 [0.02, 0.4], *P* = .031), and total language (95% CI = 0.2 [0.02, 0.4], *P* = .042) skills.

Maternal factors including higher schooling compared with no schooling had significantly positive effects on all domains of child development except motor skill. The children whose mothers completed primary schooling had significantly higher cognitive (95% CI = 0.33 [0.01, 0.63], *P* = .041), receptive communication (95% CI = 0.38 [0.09, 0.67], *P* = .01), and social–emotional (95% CI = 0.48 [0.15, 0.79], *P* = .003) skills compared with no schooling. The children of mothers with secondary and above schooling had significantly higher expressive communication (95% CI = 0.6 [0.2, 0.9], *P* = .018) and total language (95% CI = 0.5 [0.05, 0.9], *P* = .03) skills compared with mothers with no schooling. Higher maternal BMI was related to higher motor skills of the children (gross motor: 95% CI = 0.03 [0.01, 0.06], *P* = .019; total motor (95% CI = 0.03 [−0.02, 0.01], *P* = .026; results are shown in Table S2).

## 4 DISCUSSION

This study reveals that young stunted children living in urban slums of Dhaka city had poorer developmental skills relative to nonstunted children in all domains of cognitive, motor language, and social–emotional development. This is the first study in Bangladesh that reports association between stunting and the developmental level of young children over a period of time.

The present study was an observational birth cohort study; therefore compared with other observational studies, it had a less potential bias of selecting subjects for the comparison group.

The Bayley‐III that was used in the present study is a widely used valid developmental assessment tool in many low‐income countries with high interrater and test–retest reliability (Frongillo, Tofail, Hamadani, Warren, & Mehrin, 2014). The present study found an association of stunting with the developmental score, but no causality can be assigned, given the study design.Negative impacts of stunting on cognitive function have been documented in many other low‐income countries. In a Peruvian birth cohort study, children with severe stunting with LAZ < −3 in the second year of life scored 10 points lower on the revised Wechsler Intelligence Scale for Children test than the children who were not stunted (Berkman, Lescano, Gilman, Lopez, & Black, 2002). In another Peruvian study, children assessed at 6–18 months of age were followed up for 4.5–6 years, and results revealed that stunting in infancy and childhood resulted in significantly lower scores on developmental assessments compared with the nonstunted children (Crookston et al., 2010). In a birth cohort study in the Philippines, severely stunted children before 2 years of age or with persistent stunting through 8 years had significantly lower cognitive scores than the nonstunted children or children who achieved catch‐up growth (Mendez & Adair, 1999). An Indian study also reported poor performance of malnourished children in the age groups of 5–7 and 8–10 years, on tests of attention, working memory, learning and memory, and visuospatial ability (Kar et al., 2008). The cognitive scores remained unchanged even with a nutritional supplementation for 6 months to both stunted and nonstunted children in an Indian study (Sokolovic et al., 2014). One Bangladeshi study conducted by Tarleton and colleagues assessed cognitive function of school children aged 6–9 years, living in a slum of Mirpur. The cognitive scores on two verbal and two nonverbal tests (definition sections) of the Wechsler Abbreviated Scale of Intelligence and Colored Progressive Matrices found negative associations with stunting as well as the height‐for‐age and weight‐for‐age scores at study enrolment (Tarleton et al., 2006).

Motor development of a child is a process through which a child acquires movement patterns and skills. Motor skills depend on several factors such as physical and neuromuscular maturation, interaction of rearing practices, and the environment (Malina, 2004). Fine motor activities include the ability to produce precise, efficient, and adaptive movement by using small muscles of fingers, toes, lips, and tongue and coordination of eyes and hands together. Gross motor skills involve movements that use the large muscles in the arms, legs, torso, and feet. In humans, skeletal muscle is located mainly in the limbs and arms, and leg lengths are likely to be determinants of muscle mass. Similar to wasting, stunted children also been found to have decreased muscle mass due to reduced limb length, which is less clinically visible when compared with wasted children (Pomeroy et al., 2012). Epidemiological studies suggest that in stunted children, the length deficit in the legs predominates and usually occurs during the first 2 years of life, when linear growth in the lower part of the body predominates (Bogin & Varela‐Silva, 2010). In the present study, the poor motor skills of the stunted children may be due to reduced muscle mass.

Receptive and expressive communications are the two types of language skills which vary greatly, both between individuals, and within each child throughout development. Receptive language is the ability to “understand” language whereas expressive language is the ability to “communicate” using language. According to WHO conceptual framework on childhood stunting, reduced language development is one of the important short‐term consequences of early childhood stunting (Stewart, Iannotti, Dewey, Michaelsen, & Onyango, 2013). In a longitudinal cohort in Vietnam, an increase of 1*SD* in HAZ at the age of 1 year led to an increase of one fourth (24%) of a SD of language ability on the Peabody Picture Vocabulary Test at the age of 5 years (Duc, 2009). In the present study, the children who were stunted had significantly poorer communication skills compared with their nonstunted counterparts. It is known that expressive communication skills enable a child to be able to express their wants and needs, thoughts, and ideas; argue a point of view; and to interact with others through use of gestures, words, and sentence (Kid Sense Child Development, 2013). Therefore, if this delayed language development of the stunted children persists, it might have a negative impact on their future school performance and lead to other unfavourable outcomes such as higher unemployment rates, increased vulnerability to mental disorders, psychosocial problems, and overall social disadvantage (Clegg, Hollis, Mawhood, & Rutter, 2005; Goldston et al., 2004).

Similar to stunting, underweight was a strong predictor of developmental deficits in all domains except on the social–emotional scale, whereas wasting was for motor development, compared with their counterparts. A recently conducted Tanzanian cohort study with children between 18 and 36 months found negative association between malnutrition and cognitive, communication, and motor development, but found no evidence that these associations were limited to children with stunting. In this population, both HAZ and weight‐for‐age *z*‐score (WAZ) exhibited linear relation with all three developmental domains. Each unit increase in HAZ was associated with +0.09, +0.10, and +0.13, and each unit increase in WAZ was associated with +0.07, +0.10, and 0.11 higher cognitive, communication, and motor development *z*‐scores, respectively. The wasted children (defined as weight‐for‐height *z*‐score < −2) had −0.63, −0.32 and −0.54 *z*‐score deficits in cognitive, communication, and motor development, respectively, compared with nonwasted children. However, weight‐for‐height *z*‐score exhibited a nonlinear relation in contrast to HAZ and WAZ (Sudfeld et al., 2015). In the Peruvian prospective study by Berkman et al. (2002), no association was found between weight‐for‐length *z*‐score or wasting (defined as weight‐for‐length *z*‐score < −2) during infancy and Wechsler Intelligence Scale for Children‐Revised scores at 9 years of age. One Srilankan study conducted by Subasinghe and Wijesinghe (2007) reported lower cognitive abilities in the stunted children aged 36–54 months where wasted children had lower fine motor and gross motor skills, compared with normal children.

In the present study, higher post‐partum (2 months) BMI was related to higher motor development of the children. Evidence is limited on association between post‐partum BMI and child development. In the present study, positive association was found between post‐partum BMI and motor development, particularly gross motor skills of stunted and wasted children, compared with their counterparts. However, no relation of post‐partum BMI was revealed with cognitive development. It is believed that the women with preconception normal BMI (18.5–24.9 kg/m^2^) tend to gain an appropriate amount of weight during pregnancy (Institute of Medicine (US) and National Research Council (US) Committee to Reexamine IOM Pregnancy, 2009). In developing countries of Asia, women generally have a lower BMI and a smaller gestational weight gain than those reported in developed countries from Europe and North America (Xiao et al., 2017). The mean total weight gains range from 4.8 kg in Bangladesh to 10–15 kg in Europe and USA (Siega‐Riz & Adair, 1993). In our study, insufficient gestational weight gains (GWG) due to low BMI have been hypothesized to be associated with delayed motor development via inadequate fetal growth, ability level and rate of physical maturation.

Childhood stunting itself is an extremely important problem from a public health perspective. It has long‐term impact on subsequent growth and cognition. Evidence shows that stunted children complete fewer years of schooling and if they have access to education often do less well in school, which ultimately leads to fewer professional opportunities later in life and less earning (Grantham‐McGregor et al., 2007; Carba, Tan, & Adair, 2009; Thomas & Strauss, 1977; Ross et al., 2003). With an assumption of increasing adult yearly income by 9% for every year of schooling, it is estimated that 22.2% of adult income potential is lost as a result of being stunted (Grantham‐McGregor et al., 2007). Early childhood stunting resulted in poorer psychological functioning in Jamaican adolescents (Walker et al., 2005) and, in adults of 22 years, there are reports of more violent behaviours (Walker et al., 2011), and reduced formal employment at age 20–22 years in the Philippines (Carba et al., 2009). There is evidence that one unit increase in LAZ is associated with 2% higher likelihood of formal compared with informal work in the Philippines (Carba et al., 2009); an increase in height of 1% was found to be associated with a 2.4% increase in wages in Brazil (Thomas & Strauss, 1997) and reductions in child stunting resulted in future economic productivity gains of nearly 101 billion Yen in China (Ross et al., 2003). In the present study, almost half of the children had linear growth restriction at age 2 years which is alarming and requires appropriate intervention. However, there is no single strategy for treating stunting and promoting ECD. In Bangladesh few successful research trials have been conducted among rural and urban communities which integrated child development activities with nutritional interventions (Hamadani, Nahar, Huda, & Tofail, 2014).

The burden of underweight among under‐five children in Bangladesh is very high and close to stunting (33% vs. 36%; National Institute of Population Research and Training (NIPORT), Mitra and Associates, & ICF International, 2016). There is little evidence whether early childhood underweight and wasting (acute malnutrition) has any long‐term effect on child development. The results of inverse relation of underweight with developmental skills in the present study is hence of public health importance and should be given similar priority as stunting in terms of targeting for intervention programmes.

Although the global prevalence of wasting is lower than stunting and underweight, seventy percent of the world's wasted children live in Asia, most in South‐Central Asia (United Nations Children's Fund, World Health Organization, & The World Bank, 2012) where in Bangladesh the prevalence is 16% (National Institute of Population Research and Training (NIPORT), Mitra and Associates, & ICF International, 2016). These children are at substantial increased risk of severe acute malnutrition and death. From birth throughout childhood, children use motor skills to play and interact with the environment. As children grow older, deficits in gross motor skills may greatly affect play skills which is required for brain growth, physical development, communication and social growth. Pre‐school children who lack motor skills are likely to be less social, more hesitant in social interactions with peers, and more likely to be rejected by classmates (Trawick‐Smith, 2014). The poor motor development particularly gross motor skills of the wasted children of the present study, compared with the nonwasted children, might have a negative impact on their social functioning and peer relations in future.

The Government of Bangladesh is committed to reducing poverty, improving human development, and reducing inequality, and ECD has been incorporated into the national plan of action. However, the activities are not fully established in places, and there is a lack of scientific evaluation of the ECD programme. While prevalence of stunting in Bangladesh is decreasing slowly (51% in 2004 to 36% in 2014), the increasing total number under ‐5 population keeps the burden unchanged. Therefore, effective interventions in early life should target not only the reduction of stunting but also wasting and underweight. Investment in ECD programmes, particularly parenting programmes or high‐quality centre‐based care for disadvantaged children need to be implemented to yield lifetime gains and sustainable improvements in the future generations in Bangladesh. Follow‐up of these children is required to investigate the long‐term effect of stunting, underweight, and wasting on neurodevelopmental outcome. More research in different contexts is necessary to explore the effects of the intrauterine environment, micronutrient deficiencies on child development, and causality of early stunting.

## CONFLICTS OF INTEREST

The authors declare that they have no conflicts of interest.

## CONTRIBUTIONS

BN is responsible for development of concept, data analysis, and reporting of the study results. MH has contributed to the data analysis and manuscript writing. MM is one of the cosupervisors, assisted with the study design, supervised the field visits, and edited the first draft. TA is the principal investigator of the project in Bangladesh, developed the protocol, and edited the first draft. MMI, MIH, LMK, and JS edited the first draft. All authors read and approved the final manuscript.

## Supporting information


**Table S1.** Comparison of ECD scores (*z*‐scores)*on BSID‐III between underweight and not‐underweight children

**Table S2.** Comparison of ECD scores (*z*‐scores)*on BSID‐III between wasted and nonwasted children
