# Supplementary material for: Early childhood development and stunting: Findings from the MAL‐ED birth cohort study in Bangladesh
Source: Matern Child Nutr. 2019 Aug 6;16(1):e12864. doi: 10.1111/mcn.12864 (PMC7038907; doi:10.1111/mcn.12864)
Supplement: Supplementary file 1 — Table S1. Comparison of ECD scores (z‐scores)*on BSID‐III between underweight and not‐underweight children [file MCN-16-e12864-s001.docx]

**SUPPORTING MATERIALS**

**SUPPLEMENTAL TABLE 1** Comparison of ECD scores (z-scores)*on BSID-III between underweight and not-underweight children

|  | **COGNITIVE** | | | | **SOCIAL-EMOTIONAL** | | | |
| --- | --- | --- | --- | --- | --- | --- | --- | --- |
| **Predictors** | **Unadjusted**  **Coef. (95% CI)** | **P value** | **Adjusted**  **Coef. (95% CI)** | **P value** | **Unadjusted**  **Coef. (95% CI)** | **P value** | **Adjusted**  **Coef. (95% CI)** | **P value** |
| Mother's age | 0.0 (-0.01, 0.5) | 0.847 | 0.0 (-0.02, 0.03) | 0.569 | -0.02 (-0.04,0.01) | 0.183 | -0.01 (-0.0, 0.01) | 0.506 |
| Mother's BMI | 0.01 (-0.01, 0.04) | 0.352 | 0.0 (-0.03, 0.03) | 0.848 | 0.03 (-0.0, 0.06) | 0.054 | 0.02 (-0.01, 0.06) | 0.175 |
| Mother’s education | | | | |  |  |  |  |
| No schooling | Reference |  | Reference |  | Reference |  | Reference |  |
| Primary incomplete | 0.2 (-0.2, 0.5) | 0.333 | 0.1 (0.2, 0.4) | 0.437 | 0.4 (0.04, 0.7) | **0.030** | 0.3 (-0.1, 0.7) | 0.088 |
| Primary complete | 0.3 (0.03, 0.6) | **0.028** | 0.3 (-0.02, 0.6) | 0.064 | 0.05 (0.2, 0.8) | **<0.001** | 0.5 (0.1, 0.8) | **0.005** |
| HSC & above | 0.03 (-0.5, 0.5) | **0.924** | -0.0 (-0.6, 0.5) | 0.977 | 0.3 (-0.1, 0.7) | 0.207 | 0.1 (-0.3, 0.6) | 0.488 |
| Maternal depressive symptoms | -0.0 (-0.02, 0.02) | 0.841 | 0.0 (-0.01, 0.02) | 0.828 | 0.0 (-0.02, 0.02) | 0.872 | 0.0 (-0.02, 0.02) | 0.808 |
| Child's age | **-**0.0 (-0.01, 0.0) | 0.985 | 0.0 (-0.0, 0.02) | 0.267 | 0.0 (-0.01, 0.01) | 0.780 | 0.0 (-0.0, 0.01) | 0.358 |
| Child’s sex (female) | 0.1 (-0.1, 0.3) | 0.500 | 0.1 (-0.1, 0.3) | 0.282 | -0.04 (-0.2, 0.3) | 0.695 | 0.1 (-0.1, 0.3) | 0.391 |
| **Child's underweight** | -0.4 (-0.6, -0.2) | **0.001** | -0.4 (-0.6, -0.2) | **0.001** | -0.3 (-0.4, -0.1) | **0.007** | -0.2 (-0.4, -0.04) | **0.017** |

* Generalized estimating equation (GEE) analysis controlling for age and sex of the child; mother’s age, schooling, BMI and depressive symptoms

* Standard error adjusted for clustering on ID

Abbreviation:

ECD: early childhood development; BSID-III: Bayley scales of infant development, 3^rd^ version; BMI: body mass index; underweight: weight-for-age, z-score <-2SD; not-underweight: weight-for-age, z-score ≥-2SD

Mother’s education: primary incomplete (1-4^th^ grade); primary complete (5-10^th^grade); HSC=higher secondary (11-12th grade) and above (graduation and masters).

Number of observation: 637; Number of children represented: 237; Number of children represented: n=236 at 6 months, 212 at 15 months and 189 at 24 months

Reference: base score against which the others were compared

**SUPPORTING MATERIALS**

|  | **MOTOR** | | | | | | | | | | | |
| --- | --- | --- | --- | --- | --- | --- | --- | --- | --- | --- | --- | --- |
|  | **Fine motor** | | | | **Gross motor** | | | | **Total motor** | | | |
| **Predictors** | **Unadjusted**  **Coef. (95% CI)** | **P value** | **Adjusted**  **Coef. (95% CI)** | **P value** | **Unadjusted**  **Coef. (95% CI)** | **P value** | **Adjusted**  **Coef. (95% CI)** | **P value** | **Unadjusted**  **Coef. (95% CI)** | **P value** | **Adjusted**  **Coef. (95% CI)** | **P value** |
| Mother's age | 0.0 (-0.01, 0.02) | 0.737 | -0.0 (-0.0, 0.01) | 0.942 | 0.0 (-0.01, 0.03) | 0.572 | 0.0 (-0.02, 0.03) | 0.736 | 0.01 (-0.01, 0.03) | 0.631 | 0.0 (-0.02, 0.02) | 0.863 |
| Mother's BMI | 0.0 (-0.0, 0.04) | 0.090 | 0.02 (-0.0, 0.04) | 0.178 | 0.04 (0.01, 0.1) | **0.003** | 0.03 (0.0, 0.1) | **0.042** | 0.04 (0.01, 0.1) | **0.005** | 0.03 (0.0, 0.1) | **0.049** |
| Mother’s education |  |  |  |  |  |  |  |  |  |  |  |  |
| No schooling | Reference |  | Reference |  | Reference |  |  |  | Reference |  | Reference |  |
| Primary incomplete | 0.08 (-0.2, 0.3) | 0.509 | -0.02 (-0.3, 0.2) | 0.896 | 0.2 (-0.1, 0.5) | 0.262 | 0.02 (-0.3, 0.3) | 0.888 | 0.2 (-0.1, 0.5) | 0.281 | 0.02 (-0.3, 0.3) | 0.941 |
| Primary complete | 0.06 (-0.1, 0.3) | 0.563 | 0.02 (-0.2, 0.3) | 0.851 | 0.2 (0.01, 0.5) | **0.042** | 0.1 (-0.1, 0.3) | 0.380 | 0.2 (-0.02, 0.5) | 0.071 | 0.1 (0.1, 0.3) | 0.429 |
| HSC & above | -0.0 (-0.5, 0.5) | 0.977 | -0.01 (-0.6, 0.4) | 0.830 | 0.2 (-0.1, 0.5) | 0.272 | -0.03 (-0.3, 0.3) | 0.833 | 0.2 (-0.3, 0.6) | 0.464 | -0.02 (-0.4, 0.4) | 0.917 |
| Maternal depressive symptoms | -0.0 (-0.02, 0.01) | 0.458 | -0.0 (-0.02, 0.01) | 0.500 | 0.0 (-0.0, 0.01) | 0.429 | -0.0 (-0.02, 0.01) | 0.716 | -0.0 (-0.03, 0.01) | 0.421 | -0.0 (-0.02, 0.01) | 0.273 |
| Child's age | 0.0 (-0.0, 0.01) | 0.953 | 0.0 (-0.5, -0.01) | 0.478 | 0.0 (-0.01, 0.01) | .949 | 0.01 (-0.0, 0.02) | 0.148 | 0.0 (-0.01, 0.01) | 0.988 | 0.01 (-0.0, 0.01) | 0.168 |
| Child’s sex (female) | 0.2 (0.05. 0.4) | **0.010** | 0.02 (0.05, 0.4) | **0.011** | 0.02 (-0.2, 0.2) | 0.858 | 0.01 (-0.2, 0.2) | 0.896 | 0.1 (-0.09, 0-3) | 0.268 | 0.1 (-0.1, 0.3) | 0.273 |
| **Child's underweight** | -0.2 (-0.5, -0.03) | **0.020** | -0.2 (-0.4, -0.01) | **0.039** | -0.6 (-0.8, -0.3) | **<0.001** | -0.6 (-0.8, -0.4) | **<0.001** | -0.5 (-0.7, -0.3) | **<0.001** | -0.5 (-0.8, -0.3) | **<0.001** |

* Generalized estimating equation (GEE) analysis controlling for age and sex of the child; mother’s age, schooling, BMI and depressive symptoms

* Standard error adjusted for clustering on ID

Abbreviation:

BMI: body mass index; underweight: weight-for-age, z-score <-2SD; not-underweight: weight-for-age, z-score ≥-2SD

Mother’s education: primary incomplete (1-4^th^ grade); primary complete (5-10^th^grade); HSC=higher secondary (11-12th grade) and above (graduation and masters).

Number of observation: 637; Number of children represented: 237; Number of children represented: n=236 at 6 months, 212 at 15 months and 189 at 24 month

Reference: base score against which the others were compared

**SUPPORTING MATERIALS**

|  | **LANGUAGE** | | | | | | | | | | | |
| --- | --- | --- | --- | --- | --- | --- | --- | --- | --- | --- | --- | --- |
|  | **Receptive communication** | | | | **Expressive communication** | | | | **Total language** | | | |
| **Predictors** | **Unadjusted**  **Coef. (95% CI)** | **P value** | **Adjusted**  **Coef. (95% CI)** | **P value** | **Unadjusted**  **Coef. (95% CI)** | **P value** | **Adjusted**  **Coef. (95% CI)** | **P value** | **Unadjusted**  **Coef. (95% CI)** | **P value** | **Adjusted**  **Coef. (95% CI)** | **P value** |
| Mother's age | -0.01 (-0.02, 0.01) | 0.371 | -0.0 (-0.02, 0.01) | 0.664 | -0.0 (-0.02, 0.01) | 0.651 | -0.0 (-0.03, 0.01) | 0.469 | -0.0 (-0.02, 0.01) | 0.498 | -0.01 (-0.03, 0.01) | 0.517 |
| Mother's BMI | 0.02 (-0.01,0.04) | 0.236 | 0.0 (-0.02, 0.04) | 0.534 | 0.0 (-0.0, 0.04) | 0.115 | 0.01 (-0.0, 0.05) | 0.163 | 0.2 (-0.0, 0.04) | 0.132 | 0.01 (-0.01, 0.04) | 0.247 |
| Mother's education | | | | |  |  |  |  |  |  |  |  |
| No schooling | Reference |  | Reference |  | Reference |  | Reference |  | Reference |  | Reference |  |
| Primary incomplete | 0.2 (-0.1, 0.4) | 0.251 | 0.1 (-0.2, 0.4) | 0.363 | -0.01 (-0.3, 0.3) | 0.928 | -0.1 (-0.4, 0.2) | 0.410 | 0.03 (-0.2, 0.3) | 0.824 | -0.04 (-0.3, 0.2) | 0.758 |
| Primary complete | 0.4 (0.1, 0.6) | **0.006** | 0.4 (0.1, 0.7) | **0.015** | 0.2 (-0.1, 0.4) | 0.193 | 0.1 (-0.1, 0.3) | 0.481 | 0.3 (0.01, 0.5) | **0.037** | 0.2 (-0.1, 0.5) | 0.120 |
| HSC & above | 0.3 (-0.1, 0.8) | 0.178 | 0.3 (-0.2, 0.8) | 0.212 | 0.6 (0.2, 0.9) | **0.007** | 0.1 (0.02, 0.1) | **0.042** | 0.5 (0.07. 0.9) | **0.021** | 0.4 (-0.01, 0.9) | 0.053 |
| Maternal depressive symptoms | 0.01 (-0.01, 0.02) | 0.326 | 0.0 (-0.0, 0.03) | 0.106 | 0.0 (-0.01, 0.02) | 0.984 | 0.0 (-0.01, 0.02) | 0.551 | 0.0 (-0.01, 0.02) | 0.544 | 0.01 (-0.0, 0.03) | 0.247 |
| Child's age | -0.0 (-0.01, 0.01) | 0.934 | 0.0 (-0.01, 0.01) | 0.523 | -0.0 (-0.01, 0.01) | 0.981 | 0.0 (-0.0, 0.01) | 0.488 | -0.0 (-0.01, 0.0) | 0.943 | 0.0 (-0.0, 0.01) | 0.408 |
| Child’s sex (female) | 0.1 (-0.04, 0.3) | 0.136 | 0.2 (0.01,0.4) | **0.037** | 0.1 (-0.2, 0.1) | 0.414 | 0.1 (-0.1, 0.3) | 0.251 | 0.1 (-0.04, 0.3) | 0.153 | 0.1 (0.0, 0.4) | **0.047** |
| **Child's underweight** | -0.2 (-0.4, -0.04) | **0.019** | -0.2 (-0.4, 0.0) | 0.055 | -0.3 (-0.5, -0.1) | **0.012** | -0.2 (-0.4, -0.01) | **0.032** | -0.3 (-0.5, -0.1) | **0.004** | -0.3 (-0.5, -0.1) | **0.013** |

* Generalized estimating equation (GEE) analysis controlling for age and sex of the child; mother’s age, schooling, BMI and depressive symptoms

* Standard error adjusted for clustering on ID

Abbreviation:

BMI: body mass index; underweight: weight-for-age, z-score <-2SD; not-underweight: weight-for-age, z-score ≥-2SD

Mother’s education: primary incomplete (1-4^th^ grade); primary complete (5-10^th^grade); HSC=higher secondary (11-12th grade) and above (graduation and masters).

Number of observation: 637; Number of children represented: 237; Number of children represented: n=236 at 6 months, 212 at 15 months and 189 at 24 months

Reference: base score against which the others were compared
