# Supplementary material for: Early childhood development and stunting: Findings from the MAL‐ED birth cohort study in Bangladesh
Source: Matern Child Nutr. 2019 Aug 6;16(1):e12864. doi: 10.1111/mcn.12864 (PMC7038907; doi:10.1111/mcn.12864)
Supplement: Supplementary file 2 — Table S2. Comparison of ECD scores (z‐scores)*on BSID‐III between wasted and nonwasted children [file MCN-16-e12864-s002.docx]

**SUPPORTING MATERIALS**

**SUPPLEMENTAL TABLE 2** Comparison of ECD scores (z-scores)*on BSID-III between wasted and non-wasted children

|  | **COGNITIVE** | | | | **SOCIAL-EMOTIONAL** | | | |
| --- | --- | --- | --- | --- | --- | --- | --- | --- |
| **Predictors** | **Unadjusted**  **Coef. (95% CI)** | **P value** | **Adjusted**  **Coef. (95% CI)** | **P value** | **Unadjusted**  **Coef. (95% CI)** | **P value** | **Adjusted**  **Coef. (95% CI)** | **P value** |
| Mother's age | 0.0 (-0.02, 0.02) | 0.847 | 0.0 [-0.01, 0.03] | 0.525 | -0.02 (-0.04, 0.0) | 0.183 | -0.0 [-0.03, 0.01] | 0.544 |
| Mother's BMI | 0.01 (-0.01, 0.03) | 0.352 | 0.0 [-0.02, 0.03] | 0.599 | 0.03 (-0.0, 0.06) | 0.054 | 0.03 [-0.0, 0.06] | 0.132 |
| Mother’s education | | | | |  |  |  |  |
| No schooling | Reference |  | Reference |  | Reference |  | Reference |  |
| Primary incomplete | 0.12 (-0.12, 0.46) | 0.335 | 0.12 [-0.19, 0.42] | 0.465 | 0.4 (0.04, 0.7) | **0.030** | 0.29 [-0.06, 0.64] | 0.099 |
| Primary complete | 0.3 (0.03, 0.57) | **0.028** | 0.33 [0.01, 0.63] | **0.041** | 0.5 (0.24, 0.83) | **<0.001** | 0.48 [0.15, 0.79] | **0.003** |
| HSC & above | 0.02 (-0.5, 0.5) | 0.924 | 0.04 [-0.52, 0.59] | 0.883 | 0.24 (-0.15, 0.67) | 0.207 | 0.17, [-0.25, 0.59] | 0.431 |
| Maternal depressive symptoms | -0.0 (-0.02, 0.02) | 0.871 | 0.0 [-0.02, 0.02] | 0.822 | 0.0 (-0.02, 0.02) | 0.827 | 0.0 [-0.02, 0.02] | 0.911 |
| Child's age | **-**0.0 (-0.01, 0.01) | 0.985 | 0.00 [-0.0, 0.01] | 0.647 | 0.0 (-0.0, 0.0) | 0.780 | 0.0 [-0.01, 0.01] | 0.616 |
| Child’s sex (female) | 0.07 (-0.1, 0.3) | 0.500 | -0.13 [0.08, 0.34] | 0.226 | -0.4 (-0.17, 0.28) | 0.695 | -0.1[-0.1,0.3] | 0.331 |
| **Child's wasting** | -0.4 (-0.7, -0.0) | **0..048** | -0.3 [-0.66, 0.03) | 0.075 | -0.3 (-0.7, 0.09) | 0.128 | -0.25 [-0.63, 0.13] | 0.206 |

* Generalized estimating equation (GEE) analysis controlling for age and sex of the child; mother’s age, schooling, BMI and depressive symptoms

* Standard error adjusted for clustering on ID

Abbreviation:

ECD: early childhood development; BSID-III: Bayley scales of infant development, 3^rd^ version; BMI: body mass index; wasting: weight-for-length, z-score <-2SD; non-wasting: weight-for-length, z-score ≥-2SD

Mother’s education: primary incomplete (1-4^th^ grade); primary complete (5-10^th^grade); HSC=higher secondary (11-12th grade) and above (graduation and masters).

Number of observation: 637; Number of children represented: 237, Number of children represented: n=236 at 6 months, 212 at 15 months and 189 at 24 months

Reference: base score against which the others were compared

**SUPPORTING MATERIALS**

|  | **MOTOR** | | | | | | | | | | | |
| --- | --- | --- | --- | --- | --- | --- | --- | --- | --- | --- | --- | --- |
|  | **Fine motor** | | | | **Gross motor** | | | | **Total motor** | | | |
| **Predictors** | **Unadjusted**  **Coef. (95% CI)** | **P value** | **Adjusted**  **Coef. (95% CI)** | **P value** | **Unadjusted**  **Coef. (95% CI)** | **P value** | **Adjusted**  **Coef. (95% CI)** | **P value** | **Unadjusted**  **Coef. (95% CI)** | **P value** | **Adjusted**  **Coef. (95% CI)** | **P value** |
| Mother's age | 0.0 (-0.01, 0.02) | 0.737 | -0.0 [-0.02, 0.02] | 0.993 | 0.0 (-0.02, 0.02) | 0.572 | 0.0 [-0.01, 0.01] | 0.481 | 0.01 (-0.01, 0.03) | 0.631 | 0.0 [-0.02, 0.03] | 0.771 |
| Mother's BMI | 0.02 (-0.0, 0.05) | 0.090 | 0.02 [-0.0, 0.05] | 0.139 | 0.04 (0.01, 0.06) | **0.003** | 0.03 [0.01, 0.06] | **0.019** | 0.03 (0.01, 0.07) | **0.005** | 0.03 [-0.02, 0.01] | **0.026** |
| Mother’s education |  |  |  |  |  |  |  |  |  |  |  |  |
| No schooling | Reference |  | Reference |  | Reference |  |  |  | Reference |  | Reference |  |
| Primary incomplete | 0.08 (-0.2, 0.3) | 0.509 | -0.03 [-0.3, 0.2] | 0.801 | 0.2 (-0.1,0.5) | 0.262 | -0.01 [-0.3, 0.3] | 0.942 | 0.2 (-0.1, 0.5) | 0.281 | -0.02 [-0.3,0.3] | 0.867 |
| Primary complete | 0.06 (-0.1, 0.3) | 0.563 | 0.03 [-0.02, 0.3] | 0.816 | 0.2 (0.01, 0.5) | **0.042** | 0.14 [-0.0, 0.4] | 0.257 | 0.2 (-0.02, 0.5) | 0.071 | 0.12 [-0.04, 0.4] | 0.361 |
| HSC & above | -0.01 (-0.5, 0.5) | 0.977 | -0.04 [-0.6, 0.5] | 0.873 | 0.2 (-0.1, 0.5) | 0.272 | 0.01 [-0.3, 0.3] | 0.927 | 0.2 (-0.3, 0.6) | 0.089 | 0.01 [-0.4, 0.4] | 0.954 |
| Maternal depressive symptoms | -0.0 (-0.02, 0.01) | 0.428 | -0.0 [-0.02, 0.01] | 0.451 | -0.01 (-0.02, 0.01) | 0.858 | -0.01 [-0.02, 0.01] | 0.527 | -0.0 (-0.03, 0.01) | 0.423 | -0.0 [-0.02,0.01] | 0.537 |
| Child's age | 0.0 (-0.0, 0.01) | 0.953 | 0.0 [-0.0, 0.01] | 0.656 | 0.0 (-0.01, 0.01) | 0.949 | 0.0 [-0.0, 0.01] | 0.481 | 0.0 (-0.01, 0.01) | 0.988 | 0.0 [-0.0, 0.01] | 0.460 |
| Child’s sex (female) | 0.2 (0.05. 0.4) | **0.010** | 0.24 [0.06,0.4] | **0.007** | 0.02 (-0.2, 0.2) | 0.858 | 0.05 [-0.2, 0.3] | 0.611 | 0.1 (-0.09, 0.3) | 0.268 | 0.15 [-0.05, 0.4] | 0.154 |
| **Child's wasting** | 0.4 (-0.6, -0.1) | **0.005** | -0.37 [-0.6, -0.1] | **0.006** | -0.77 (-1.2, 0.4) | **<0.001** | -0.73 [-1.1, -0.4] | **<0.001** | -0.8 (-1.1, -0.5) | **<0.001** | -0.77 [-1.1, -0.4] | **<0.001** |

* Generalized estimating equation (GEE) analysis controlling for age and sex of the child; mother’s age, schooling, BMI and depressive symptoms

* Standard error adjusted for clustering on ID

Abbreviation:

BMI: body mass index; wasting: weight-for-length, z-score <-2SD; non-wasting: weight-for-length, z-score ≥-2SD

Mother’s education: primary incomplete (1-4^th^ grade); primary complete (5-10^th^grade); HSC=higher secondary (11-12th grade) and above (graduation and masters).

Number of observation: 637; Number of children represented: 237; Number of children represented: n=236 at 6 months, 212 at 15 months and 189 at 24 months

Reference: base score against which the others were compared

**SUPPORTING MATERIALS**

|  | **LANGUAGE** | | | | | | | | | | | |
| --- | --- | --- | --- | --- | --- | --- | --- | --- | --- | --- | --- | --- |
|  | **Receptive communication** | | | | **Expressive communication** | | | | **Total language** | | | |
| **Predictors** | **Unadjusted**  **Coef. (95% CI)** | **P value** | **Adjusted**  **Coef. (95% CI)** | **P value** | **Unadjusted**  **Coef. (95% CI)** | **P value** | **Adjusted**  **Coef. (95% CI)** | **P value** | **Unadjusted**  **Coef. (95% CI)** | **P value** | **Adjusted**  **Coef. (95% CI)** | **P value** |
| Mother's age | -0.01 (-0.02,0.01) | 0.371 | -0.0 [-0.02, 0.02] | 0.711 | -0.0 (-0.02, 0.01) | 0.651 | -0.0 [-0.03, 0.01] | 0.533 | -0.01 (-0.02, 0.01) | 0.498 | -0.1 [-0.3, 0.2] | 0.585 |
| Mother's BMI | 0.02 (-0.01, 0.04) | 0.236 | 0.0 [-0.02,0.04] | 0.427 | -0.01 (-0.3, 0.3) | 0.115 | 0.02 [-0.0, 0.05] | 0.077 | 0.02 (-0.01, 0.04) | 0.132 | 0.02 [-0.0, 0.05] | 0.135 |
| Mother's education | | | | |  |  |  |  |  |  |  |  |
| No schooling | Reference |  | Reference |  | Reference |  | Reference |  | Reference |  | Reference |  |
| Primary incomplete | 0.6 (-0.1, 0.4) | 0.251 | 0.13 [-0.16, 0.4] | 0.369 | -0.01 (-0.3, 0.3) | 0.928 | -0.1 [-0.4, 0.2] | 0.476 | 0.03 (-0.2, 0.3) | 0.824 | -0.0 [-0.3, 0.2] | 0.790 |
| Primary complete | 0.4 (0.1, 0.6) | **0.006** | 0.38 [0.09, 0.67] | **0.010** | 0.2 (-0.1, 0.4) | 0.193 | 0.13 [-0.12, 0.4] | 0.320 | 0.3 (0.02, 0.5) | **0.037** | 0.3 [-0.01, 0.5] | 0.061 |
| HSC & above | 0.3 (-0.1, 0.8) | 0.178 | 0.3 [-0.16, 0.83] | 0.182 | 0.6 (0.2, 0.9) | **0.007** | 0.5 [0.09, 0.9] | **0.018** | 0.5 (0.01, 0.9) | **0.021** | 0.5 [0.05, 0.9] | **0.030** |
| Maternal depressive symptoms | 0.01 (-0.01, 0.02) | 0.326 | 0.0 [0.0, 0.03] | 0.142 | 0.0 (-0.01, 0.02) | 0.984 | 0.0 [-0.01, 0.02] | 0.164 | 0.5 (0.1, 0.9) | **0.021** | 0.0 [0.0, 0.03] | 0.262 |
| Child's age | -0.0 (-0.0, 0.01) | 0.934 | 0.0 [-0.0, 0.01] | 0.744 | -0.0 (-0.01, 0.01) | 0.981 | 0.0 [-0.03, 0.01] | 0.871 | -0.0 (-0.01, 0.01) | 0.943 | 0.0 [-0.0, 0.01] | 0.762 |
| Child’s sex (female) | 0.1 (-0.04, 0.3) | 0.136 | 0.2 [0.02, 0.4] | **0.031** | 0.1 (-0.0 (0.3) | 0.414 | 0.1 [-0.1, 0.3] | 0.258 | -0.1 (-0.05, 0.3) | 0.153 | 0.2 [0.0, 0.4] | **0.042** |
| **Child's wasting** | -0.2 (-0.5, 0.1) | 0.133 | -0.17 [-0.46, 0.11] | 0.244 | 0.05 (-0.3, 0.4) | 0.746 | 0.1 [-0.2, 0.4] | 0.521 | -0.1 (-0.3, 0.2) | 0.508 | -0.06 [-0.3, 0.2] | 0.713 |

* Generalized estimating equation (GEE) analysis controlling for age and sex of the child; mother’s age, schooling, BMI and depressive symptoms

* Standard error adjusted for clustering on ID

Abbreviation:

BMI: body mass index; wasting: weight-for-length, z-score <-2SD; non-wasting: weight-for-length, z-score ≥-2SD

Mother’s education: primary incomplete (1-4^th^ grade); primary complete (5-10^th^grade); HSC=higher secondary (11-12th grade) and above (graduation and masters).

Number of observation: 637; Number of children represented: 237; Number of children represented: n=236 at 6 months, 212 at 15 months and 189 at 24 months

Reference: base score against which the others were compared
